# Mechanism of attenuation of leptin signaling under chronic ligand stimulation

**DOI:** 10.1186/1471-2091-11-2

**Published:** 2010-01-08

**Authors:** Holger Knobelspies, Julia Zeidler, Paul Hekerman, Simone Bamberg-Lemper, Walter Becker

**Affiliations:** 1 Institute of Pharmacology and Toxicology, Medical Faculty of the RWTH Aachen University, Wendlingweg 2, 52074 Aachen, Germany

## Abstract

**Background:**

Leptin is an adipocyte-derived hormone that acts via its hypothalamic receptor (LEPRb) to regulate energy balance. A downstream effect essential for the weight-regulatory action of leptin is the phosphorylation and activation of the latent transcription factor STAT3 by LEPRb-associated Janus kinases (JAKs). Obesity is typically associated with chronically elevated leptin levels and a decreased ability of LEPRb to activate intracellular signal transduction pathways (leptin resistance). Here we have studied the roles of the intracellular tyrosine residues in the negative feedback regulation of LEPRb-signaling under chronic leptin stimulation.

**Results:**

Mutational analysis showed that the presence of either Tyr985 and Tyr1077 in the intracellular domain of LEPRb was sufficient for the attenuation of STAT3 phosphorylation, whereas mutation of both tyrosines rendered LEPRb resistant to feedback regulation. Overexpression and RNA interference-mediated downregulation of suppressor of cytokine signaling 3 (SOCS3) revealed that both Tyr985 and Tyr1077 were capable of supporting the negative modulatory effect of SOCS3 in reporter gene assays. In contrast, the inhibitory effect of SOCS1 was enhanced by the presence of Tyr985 but not Tyr1077. Finally, the reduction of the STAT-phosphorylating activity of the LEPRb complex after 2 h of leptin stimulation was not accompanied by the dephosphorylation or degradation of LEPRb or the receptor-associated JAK molecule, but depended on Tyr985 and/or Tyr1077.

**Conclusions:**

Both Tyr985 and Tyr1077 contribute to the negative regulation of LEPRb signaling. The inhibitory effects of SOCS1 and SOCS3 differ in the dependence on the tyrosine residues in the intracellular domain of LEPRb.

## Background

Leptin is an adipocyte-secreted hormone that acts on hypothalamic centers in the brain to control the energy balance of the body [1]. Leptin deficiency of humans or rodents results in hyperphagia and morbid obesity. In patients with genetic leptin deficiency, therapy with exogenous leptin effectively suppresses hyperphagia and corrects metabolic and other abnormalities [2]. However, in most cases of obesity levels of circulating leptin are high, implying a state of resistance to the weight-reducing effect of leptin. Consequently, clinical trials using recombinant leptin for the pharmacological treatment of obesity yielded disappointing results [3,4]. Leptin resistance in rodents can be induced by short-term voluntary overfeeding [5], by feeding high-fat diet [6-9], and by chronically elevated leptin levels [10-12]. Reduced leptin sensitivity is also a physiological mechanism to allow anticipatory energy intake and storage of nutrients, *e.g*. during pregnancy or in hibernators [13]. Mechanisms of leptin resistance include failure of circulating leptin to reach its targets in the brain, inhibition of the intracellular leptin signaling cascade, endoplasmic reticulum stress, and antagonism of the physiological actions of leptin downstream from the primary target cell of leptin [14-19]. Regardless of the relative contributions of these mechanisms, it is clear that the ability of leptin to activate intracellular signaling pathways is decreased by high chronic blood levels of leptin [15]. However, the mechanisms underlying the leptin-induced state of reduced leptin sensitivity are not yet understood at the molecular level.

The leptin receptor belongs to the cytokine receptor superfamily and exists in several splicing variants. Leptin binding to the long leptin receptor isoform (LEPRb) activates cytokine-like signal transduction *via *the Janus kinase/signal transducer and activator of transcription (JAK/STAT) pathway. Leptin stimulation leads to the activation of JAKs that are constitutively associated with LEPRb and in turn phosphorylate tyrosine residues in the intracellular domain of the receptor [20]. The phosphorylated tyrosines (pTyr) provide docking sites for proteins with Src homology 2 (SH2) domains. pTyr985 (numbering refers to the sequence of murine LEPRb) recruits the tyrosine phosphatase SHP2, which mediates the activation of the RAS/RAF/ERK pathway, whereas the STAT proteins bind to pTyr1077 (STAT5) and pTyr1138 (STAT1, STAT3 and STAT5) (for recent reviews see [16,17,21]). The bound STAT factors are phosphorylated by the receptor-associated JAK kinases, dimerize and translocate to the nucleus to control transcription of specific target genes. LEPRb-induced STAT3 activation is essential for leptin regulation of energy balance [22,23].

Although SHP2 has tyrosine phosphatase activity, its overexpression or knockdown only marginally alters the levels of leptin-induced STAT3 phosphorylation [24,25]. Multiple studies support roles for two inhibitory proteins, suppressor of cytokine signaling 3 (SOCS3) and protein tyrosine phosphatase 1B (PTP1B), as negative regulators of leptin signaling. The level of PTP1B expression modulates LEPRb signaling in cell lines and *in vivo *[26], and its upregulation by leptin implies a function as a negative feedback regulator [27]. Numerous studies have demonstrated that the upregulation of SOCS3 by leptin contributes to the attenuation of LEPRb signaling *in vivo *and in cultured cells [15,28,29]. SOCS3 suppresses cytokine signaling by inhibiting the receptor-associated JAK kinase [30] or by targeting bound signaling proteins for proteasomal degradation [28,31,32]. Optimal inhibition of JAKs occurs when SOCS3 is recruited to cytokine receptor complexes *via *binding to specific phosphotyrosine motifs (pTyr759 in gp130, pTyr985 in LEPRb, pTyr401 in the erythropoietin receptor, pTyr800 in the IL-12Rβ2 [33-37]. SOCS1 is structurally similar to SOCS3 and is generally thought to inhibit JAKs by direct binding to the activation loop of the kinase, independent of receptor-tyrosine motifs [33,34,38].

Numerous studies have implicated the intracellular tyrosine residues of LEPRb in the negative regulation of leptin signaling. Tyr1138 is essential for the STAT3-dependent upregulation of the feedback inhibitor SOCS3 in response to leptin stimulation [39-41], and the mutation of Tyr1138 prevented the attenuation of ERK activation during prolonged leptin stimulation [25]. Moreover, the phenotype of knock-in mice expressing a LEPRb mutant for Tyr1138 provides evidence of increased effects of leptin that are independent of STAT3 [25]. No clear picture has yet emerged concerning the roles of Tyr985 and Tyr1077 in the attenuation of LEPRb signaling. Bjørbaek *et al*. [36] demonstrated that SOCS3 inhibits leptin signaling *via *binding to pTyr985, but the attenuation of LEPRb-induced ERK activation was later shown to occur independently of Tyr985 [25]. Tyr1077 was only recently established as an *in vivo*-phosphorylation site of LEPRb [42] although its possible role as recruitment site for SOCS3 has already been suggested in an earlier study [41]. In addition to the unresolved contribution of Tyr985 and Tyr1077 in the negative regulation of leptin signaling, the molecular mechanisms by which SOCS3 inhibits LEPRb under chronic stimulation remain poorly characterized.

In the present study we analyzed the molecular mechanisms of attenuation of leptin signaling under conditions of continuous stimulation. In particular, we focused on the contribution of the intracellular tyrosine residues in LEPRb and their interaction with SOCS1 and SOCS3. We found that the presence of one of the two proximal intracellular tyrosine residues (Tyr985 or Tyr1077) was sufficient for the attenuation of STAT3 activation, and that either tyrosine residue can support the suppression of LEPRb signaling by SOCS3. Finally, we show that the receptor-associated JAK kinase has reduced STAT-phosphorylating activity after two hours of continuous receptor stimulation. However, the kinase is not dephosphorylated or degraded, and the inhibition of activity depends on the presence of Tyr985 and Tyr1077 in LEPRb.

## Results

### Attenuation of leptin-induced STAT3 activation depends on the presence of either Tyr985 or Tyr1077

**Figure 1:**
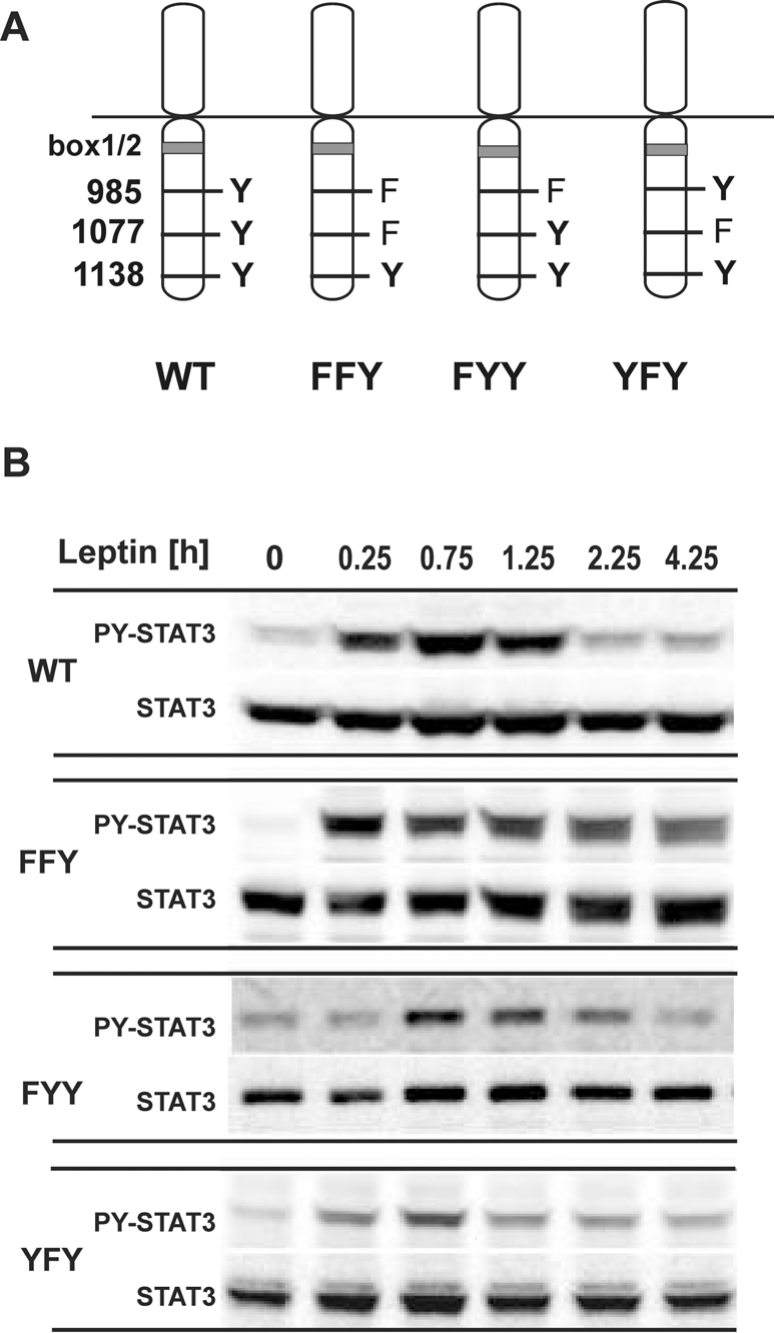
**Time course of STAT3 activation by LEPRb tyrosine/phenylalanine mutants**. **A**, Schematic representation of the LEPRb point mutants used in this study. The membrane-proximal box1/2 region binds the JAK family kinase and Tyr1138 (box3) mediates the activation of STAT3. **B**, RINm5F cells expressing WT or mutant LEPRb (FFY, FYY, YFY) were treated with leptin (20 ng/ml) for the times indicated (0 min, 15 min, 45 min, 75 min, 135 min, 255 min). Total cellular lysates were subjected to Western blot analysis and immunodetection with the indicated antibodies. These results are representative of 2 (YFY) or at least 3 (WT, FFY, FYY) independent experiments.

To examine the role of Tyr985 and Tyr1077 in the attenuation of LEPRb signaling during prolonged ligand stimulation, we determined the kinetics of leptin-induced STAT3 tyrosine phosphorylation in RINm5F cells. We have previously established the insulinoma cell line RINm5F as a model system to define the differential effects of the intracellular tyrosines in LEPRb signal transduction and their effects on gene expression [43,44]. We used RINm5F subclones expressing wild type (WT) LEPRb or mutant receptor construct in which one or both of these tyrosines were mutated to phenylalanine. The presence of Tyr1138 in the mutant receptors allowed us to monitor STAT3 tyrosine phosphorylation as the principle downstream signaling event. As shown in Figure 1, continuous treatment of RINm5F-LEPRb-WT cells with leptin resulted in a maximal tyrosine phosphorylation of STAT3 from 15 - 75 min, after which the level of the phosphorylated STAT3 declined sharply but remained detectable at reduced levels for 3 h. Mutation of neither Tyr985 nor Tyr1077 in LEPRb-FYY and LEPRb-YFY abrogated the attenuation of LEPRb signaling. STAT3 activation by the LEPRb double mutant lacking both Tyr985 and Tyr1077 (LEPRb-FFY) did not decline during the 4-h treatment with leptin. Similar time courses of STAT3 phosphorylation were observed in HIT-T15 cells transiently expressing wild type and mutant LEPRb constructs (see additional file 1: supplementary Figure S1).

### Leptin induces expression of SOCS3 but not SOCS1 in RINm5F cells

**Figure 2:**
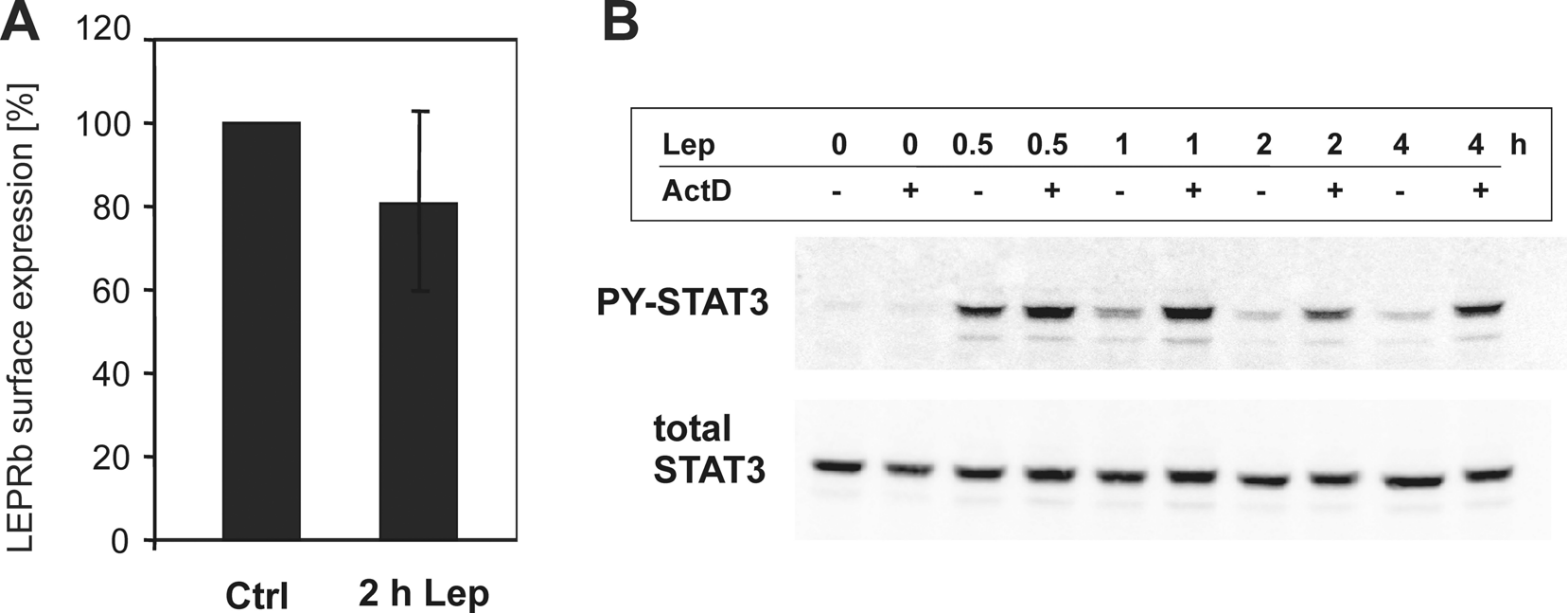
**Attenuation of LEPRb signaling is not due to receptor internalization and depends on active gene expression**. **A**, Binding of leptin-SEAP to RINm5F cells stably expressing LEPRb was determined after cells were treated with leptin (20 ng/nl) for 2 h or were not treated (Ctrl). Nonspecific binding was determined by incubating parallel cultures with leptin-SEAP in the presence of excess unlabeled leptin (2 μg/ml) and was subtracted. Bound phosphatase activity was determined using a chemoluminescence-based assay. Bars represent means and S.D. values of six independent experiments. Reduction of LEPRb surface expression after treatment with leptin was not significant (paired t-test). **B**, RINm5F cells stably expressing wild type LEPRb were treated with actinomycin D (5 μg/ml) for 30 min before stimulation with leptin (20 ng/ml). The time course of leptin-induced STAT3 phosphorylation was analyzed by Western blots of total cellular lysates with the indicated antibodies. This results are representative of two independent experiments.

**Figure 3:**
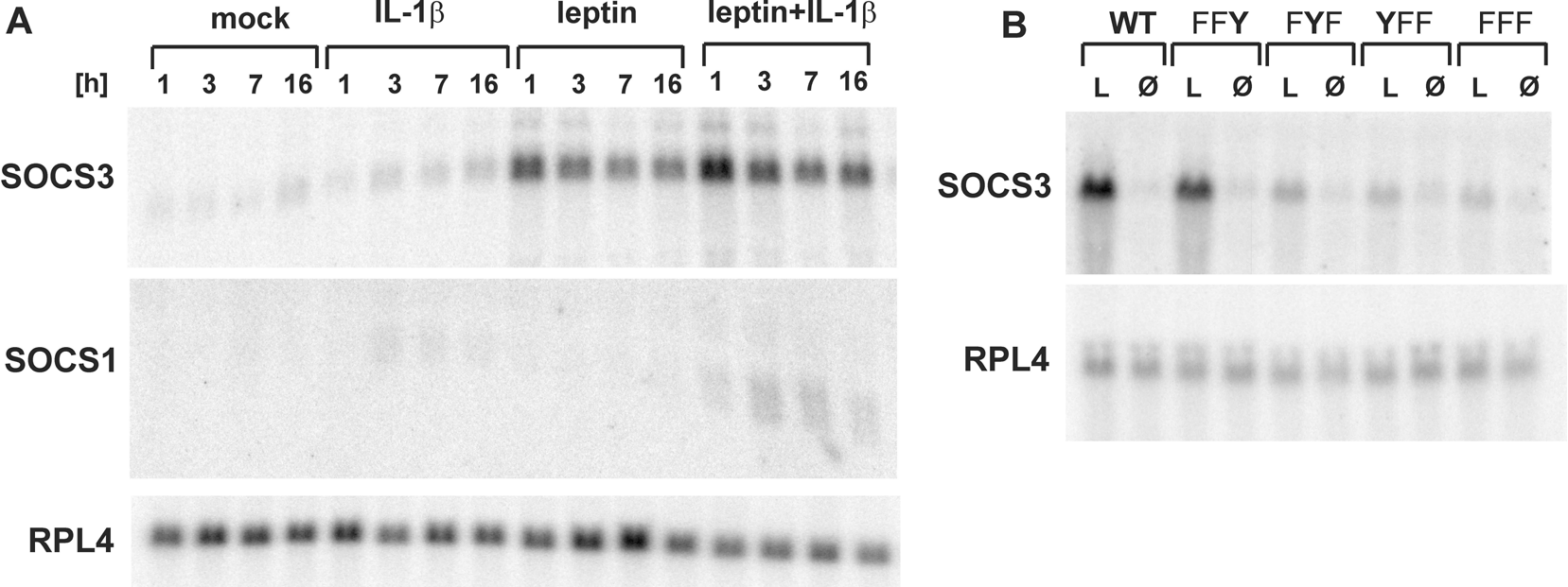
**Induction of SOCS1 and SOCS3 mRNA by leptin and IL-1β**. **A**, RINm5F cells stably expressing wild type LEPRb were treated with leptin (100 ng/ml), IL-1β (50 U/ml) or a combination of both for the times indicated (1, 3, 7 or 16 h). **B**, RINm5F cells stably expressing the indicated LEPRb tyrosine mutants were treated with 100 ng/ml leptin (L) or vehicle (Ø) for 1 h. Total RNA was isolated and subjected to Northern blot analysis with cDNA probes for SOCS1, SOCS3 or ribosomal protein L4 (RPL4) as an unregulated control mRNA.

We determined the surface expression of LEPRb in untreated and leptin-treated RINm5F cells to examine whether leptin signaling was downregulated by receptor internalization and/or degradation. After 2 h of leptin treatment, binding assays showed only a weakly reduced leptin binding to the cell surface, which could not account for the reduced STAT3 phosphorylation (Figure 2A). Next we used the transcriptional inhibitor, actinomycin D, to reveal whether active gene expression was required for feedback inhibition. As shown in Figure 2B, treatment with actinomycin D prevented the decrease of leptin-induced STAT3 phosphorylation over time. A similar result was obtained by treatment with cycloheximide (10 μg/ml; data not shown). These observations are consistent with the hypothesis that LEPRb signaling in RINm5F cells is attenuated by the upregulation of SOCS family proteins. Therefore, we examined whether leptin induced the expression of SOCS1 and SOCS3 in RINm5F cells. As previously observed in other cell lines (CHO, PC12, and 32D, rat skeletal muscle cells [39-41,45]), leptin induced a rapid upregulation of SOCS3 mRNA levels within 1 h but did not affect the expression of SOCS1 (Figure 3A). However, SOCS1 mRNA was upregulated in response to IL-1β or the simultaneous application of leptin and IL-1β. We tested this combination of cytokines because we had previously shown that IL-1β enhanced the expression of several leptin-induced genes in RINm5F cells [44]. In contrast to a previous study in RINm5F cells [46], we observed no upregulation of SOCS3 mRNA by IL-1β. Consistent with previous reports that the transcription of the SOCS3 gene is regulated by STAT3, Tyr1138 was sufficient for the induction of SOCS3 expression in RINm5F cells (Figure 3B). Thus, the LEPRb constructs used throughout this study were fully capable of inducing the expression of SOCS3.

### Inhibition of LEPRb point mutants by SOCS3 and SOCS1

**Figure 4:**
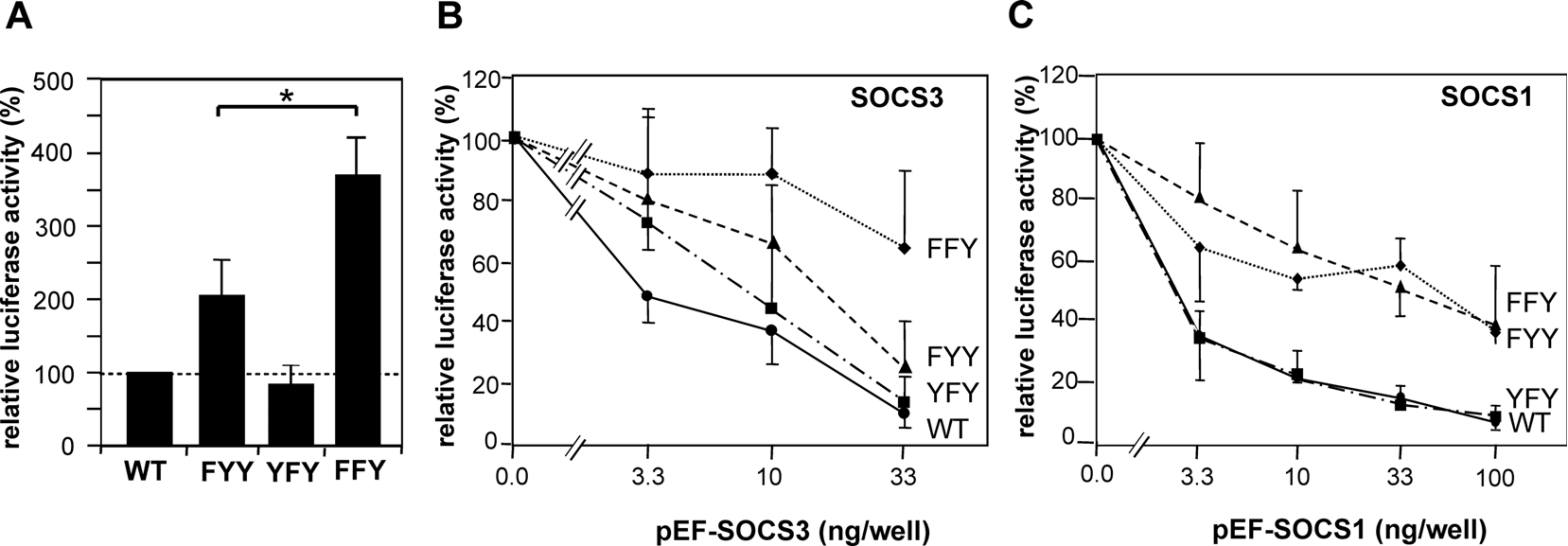
**Inhibitory effect of SOCS1 and SOCS3 on reporter gene induction by LEPRb point mutants**. HepG2 cells were transfected with pGL3α_2_M-215Luc and vectors for the indicated LEPRb point mutants along with varying amounts of expression plasmids for SOCS3 or SOCS1. Cells were treated with leptin (100 ng/ml) for 16 h before luciferase activities were determined and normalized to β-galactosidase. **A**, Reporter gene activities for the different LEPRb constructs in the absence of co-transfected pEF-SOCS expression plasmids (means ± S.D. from 6 experiments). The difference between FYY and FFY is statistically significant (Student's t-test, *P *< 0.01). **B and C**, The graphs present the normalized luciferase activities relative to those of cells expressing no exogenous SOCS protein (means ± S.D. from 3 experiments). The differential response of FYY and FFY to the highest expression level of SOCS3 was statistically significant. The difference of the response of WT and FYY to SOCS1 was significant at all expression levels (Welch's t-test, *P *< 0.05).

Next we analyzed the roles of Tyr985 and Tyr1077 in the negative regulation of LEPRb signaling with the help of STAT3-dependent reporter gene assays. These assays were performed in the hepatoma cell line HepG2 to allow for ensuing knockdown experiments with shRNA vectors directed against human SOCS1 and SOCS3 (see below). As shown in Figure 4A, mutation of Tyr985 (LEPRb-FYY) caused a twofold increase of leptin-induced reporter gene activity, consistent with the known function of this tyrosine as a docking site for SOCS3. Significantly higher luciferase activity was induced by the mutant receptor lacking both Tyr985 and Tyr1077 (FFY), indicating that Tyr1077 also plays a role in the negative regulation of LEPRb→STAT3 signaling. However, mutation of Tyr1077 alone did not affect promoter activity (YFY mutant).

To determine the requirement of Tyr985 and Tyr1077 for the inhibitory effects of SOCS proteins, we studied the effects of SOCS3 and SOCS1 overexpression on the activation of the STAT3-dependent promoter by the LEPRb mutants. Both SOCS proteins were expressed from the same vector and at comparable levels (see additional file 1: supplementary Figure S2C), allowing us to directly compare the inhibitory potencies of SOCS1 and SOCS3 on leptin-induced promoter activation. The expression level of the SOCS proteins was carefully titrated to attain a submaximal degree of inhibition.

Transfection of SOCS3 expression plasmid resulted in a dose-dependent decrease of luciferase activity induced by wild type LEPRb in HepG2 cells (Figure 4B). Mutation of Tyr985 in LEPRb-FYY reduced but did not prevent the inhibitory effect of SOCS3. In contrast, LEPRb-FFY was largely resistant to SOCS3 overexpression and was only weakly inhibited by the highest expression levels of SOCS3. This result provides evidence that SOCS3 inhibits LEPRb signaling not only by binding to Tyr985 but also to Tyr1077. A similar result was obtained in the hamster insulinoma cell line HIT-T15, although LEPRb-FYY and LEPRb-FFY differed in their sensitivity to SOCS3 only at higher expression levels (see additional file 1: supplementary Figure S2A).

Overexpressed SOCS1 inhibited wild type LEPRb as efficiently as SOCS3 (Figure 4C). Unexpectedly, SOCS1 had a significantly weaker inhibitory effect on LEPRb-FYY than on the wild type receptor, suggesting that SOCS1 exerts its effect partially by binding to pTyr985. In contrast to SOCS3, SOCS1 inhibited LEPRb-FYY and LEPRb-FFY with the same potency.

### Knockdown experiments

**Figure 5:**
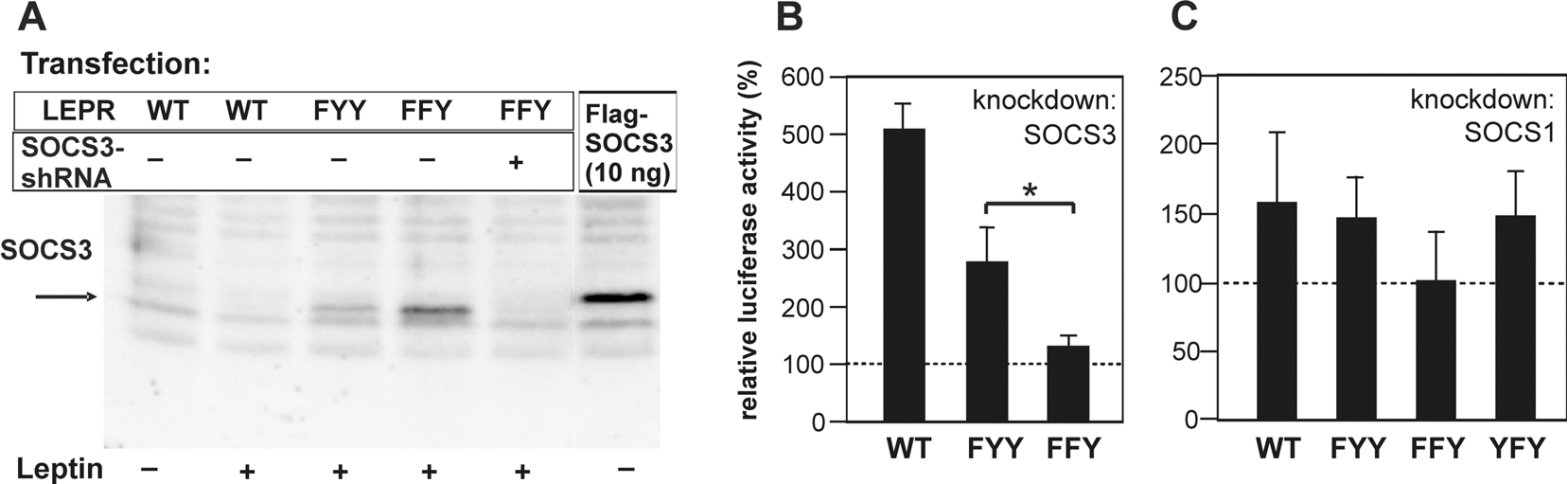
**Effect of SOCS3 knockdown on reporter gene induction by LEPRb point mutants**. **A, **SOCS3 expression levels. - HEPG2 cells were transfected with the expression plasmids for the indicated LEPRb constructs, pSUPER-SOCS3 (300 ng/well) or pEF-SOCS3 (10 ng/well). Levels of endogenous SOCS3 in leptin-treated cells (16 h, 100 ng/ml) or overexpressed FLAG-SOCS3 were determined by immunoprecipitation and Western blot analysis with SOCS3-specific antibody. **B and C**, Knockdown of SOCS1 or SOCS3. The reporter gene assays were performed as in Figure 3 except that shRNA vectors specific for SOCS1 or SOCS3 were co-transfected (300 ng/well). Columns reflect luciferase activities relative to the activity in cells transfected with empty knockdown vector (means ± S.D., n = 3 independent experiments in **B **and n = 5 in **C**). The difference of the responses of FYY and FFY to the knockdown of SOCS3 was significant (Student's t-test, *P *< 0.05).

We performed knockdown experiments to address whether endogenous levels of SOCS3 suffice to inhibit LEPRb signaling *via *binding to pTyr1077. Endogenous SOCS3 accumulated to higher levels after stimulation of LEPRb-FFY than LEPRb-FYY, confirming that Tyr1077 contributes to the negative regulation of the effects of leptin on gene expression. Expression of a SOCS3-directed shRNA reduced the expression of leptin-induced SOCS3 below the limits of detection (Figure 5A). Promoter activation by wild type LEPRb was enhanced 5-fold by knockdown of SOCS3 expression (Figure 5B), indicating that the negative feedback through SOCS3 plays a critical role in the modulation of the leptin response in HepG2 cells. Promoter activation by LEPRb-FYY was also significantly enhanced by knockdown of SOCS3, indicating that Tyr985 is not essential for the inhibitory effect of SOCS3. In contrast, the activity of LEPRb-FFY was not altered by the knockdown plasmid, suggesting that Tyr1077 must be responsible for the inhibitory effect of SOCS3 on LEPRb-FYY. Knockdown of SOCS1 expression increased LEPRb-induced promoter activity only by approximately 50% (Figure 5C) since SOCS1 is not induced by leptin (see above, Figure 3). Consistent with the above observation that the effect of SOCS1 was partially dependent on receptor tyrosine motifs, knockdown of SOCS1 did not further enhance the activity of LEPRb-FFY. However, the difference between the mutant receptor construct did not reach statistical significance.

### JAK2 inhibition during downregulation of LEPRb signaling is not correlated with dephosphorylation

**Figure 6:**
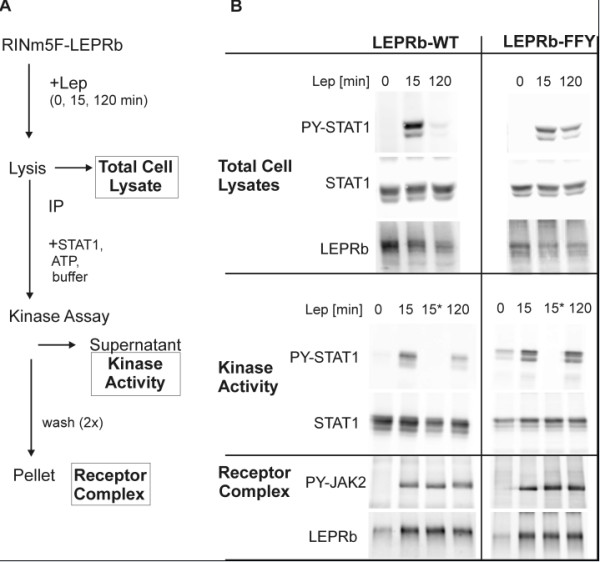
**Assay of JAK2 activity in LEPRb complexes**. **A**, Outline of the method. - RINm5F cells stably expressing WT or mutant (FFY) LEPRb were treated with leptin (20 ng/ml) for the 15 min or 120 min as indicated. LEPRb receptor complexes were immunoprecipitated (*IP*) with an antibody directed against the C-terminal myc tag of LEPRb. Immunoprecipitates were incubated with recombinant STAT1 as substrate in the presence of ATP, and phosphorylation of STAT1 was determined by Western blot analysis of the supernatant (*kinase activity*). **B**, Western blot analysis. - Activation loop phosphorylation of JAK2 was assessed in the immunoprecipitates with a phosphospecific antibody (anti Tyr1007/1008, PY-JAK2). Catalytic activity of receptor-bound JAK2 was determined as the phosphorylation of STAT1 on Tyr701 (PY-STAT1). LEPRb and total STAT were detected to control for comparable levels of expression or immunoprecipitation, respectively. A control reaction that was performed in the absence of ATP is marked by an asterisk (*). In two independent experiments, STAT1 phosphorylation by LEPRb-WT complexes was reduced by 57.3% and 42.6% after 2 h of leptin treatment, whereas the STAT1 phosphorylating activity of LEPRb-FFY complexes remained constant (103.1% and 111.3%).

SOCS1 and SOCS3 harbor KIR domains that are thought to suppress JAK kinase activity by acting as substrate mimetics [30]. We designed an assay to determine the STAT-phosphorylating activity of the JAK kinase in LEPRb receptor complexes. Immunoprecipitated receptor complexes were incubated with recombinant STAT1 in the presence of ATP, and tyrosine phosphorylation of STAT1 was determined by Western blot analysis with an antibody specific for pTyr701 (Figure 6A). STAT1 was used as a substrate because recombinant STAT3 was not commercially available at that time, and because STAT1 is also activated by leptin in RINm5F cells [44]. Similar to STAT3, tyrosine phosphorylation of the endogenous STAT1 in the lysates of the RINm5F-(LEPRb-WT) cells was reduced to near background levels after 2 h of leptin treatment but remained detectable in the LEPRb-FFY expressing cells (Figure 6B). Surprisingly, the phosphorylation of the tyrosines in the activation loop of JAK2 (Tyr1007/1008) was not reduced at this time point, and no difference in JAK phosphorylation between WT and mutant LEPRb was observed. However, STAT1-phosphorylating activity of the WT LEPRb complex was markedly reduced after 2 h leptin treatment. In contrast, kinase activity remained constant in the LEPRb-FFY complexes. This result suggests that i) LEPRb signaling during chronic receptor stimulation is downregulated at the level of JAK activity, ii) kinase activity does not correlate with the phosphorylation of the tyrosines in the activation loop of the receptor-associated JAK kinase, and iii) the downregulation of leptin signaling depends on the presence of Tyr985 and/or Tyr1077 in LEPRb.

## Discussion

In the present study we have deciphered the roles of the intracellular tyrosines for the attenuation of LEPRb signaling, in particular for the inhibitory effects of SOCS3 and SOCS1, and provide new evidence regarding the mechanism by which LEPRb signaling is terminated during chronic stimulation.

### Role of Tyr1077

We present a series of results documenting that Tyr1077 has a role in the negative regulation of LEPRb: (i) The presence of either Tyr985 or Tyr1077 in the cytoplasmic domain of LEPRb is sufficient for the attenuation of leptin-induced STAT3 phosphorylation (Figure 1). (ii) LEPRb-FFY induces significantly higher reporter gene activity in HepG2 cells than LEPRb-FYY (Figure 3A), suggesting that Tyr1077 contributes to negative regulation of leptin-induced gene expression. A similar effect has been observed in the neuronal PC12 cell line [47] but neither in Hek293 cells [41] nor our previous study of HIT-T15 insulinoma cells [43]. The cell type specificity may be due to varying expression levels of other signaling molecules that compete for binding to pTyr1077, such as STAT5, SOCS2 or CIS [43,48]. (iii) The inhibitory effect of SOCS3 was reduced but still clearly present in the absence of Tyr985 (LEPRb-FYY), whereas the mutation of both Tyr985 and Tyr1077 (LEPRb-FFY) rendered LEPRb largely resistant to the inhibitory effect of SOCS3 (Figure 3B). This result contrasts with that of Bjørbaek *et al*. [36], which suggested that overexpressed SOCS3 failed to inhibit STAT3-dependent reporter gene activity mediated by a Tyr985 mutant LEPRb construct. This difference may be attributed to the use of erythropoietin receptor-LEPRb chimeras in that study, or more likely, to the different cell systems used. Consistent with the results obtained in 293 cells by Bjørbaek *et al*. [36], we show that SOCS3 inhibited LEPRb-FYY in HIT-T15 cells only at the highest levels of expression (see additional file 1: supplementary Figure S2). (iv) The knockdown experiments show clearly that endogenous levels of SOCS3 are sufficient for the inhibition of LEPRb→STAT3 signaling *via *pTyr1077 in HepG2 cells (Figure 4B). Whereas reporter gene activity stimulated by LEPRb-FYY was significantly enhanced by knockdown of SOCS3, this was not the case for the double mutant (LEPRb-FFY). Thus, the inhibitory effect of endogenous SOCS3 is strictly dependent on the presence of either Tyr985 or Tyr1077. (v) Finally, LEPRb-FFY induced higher levels of SOCS3 than LEPRb-FYY (Figure 4A), confirming that the enhanced STAT3 activation translates into increased expression of an endogenous gene, not just the exogenous reporter construct.

Taken together, these results establish a role of pTyr1077 in the negative regulation of the LEPRb receptor complex, thus extending the prevailing view that Tyr985 is critical for the negative feedback regulation of leptin signaling. De Souza *et al*. [49] have determined affinities of 8.3 μM and 26 μM for the binding of SOCS3 to phosphopeptides corresponding to regions surrounding and Tyr985 and Tyr1077 of LEPRb. Binding to pTyr1077 in LEPRb was thus somewhat weaker than to pTyr985, much weaker than to pTyr759 in the gp130 receptor (0.1 μM), but stronger than to the SOCS3 binding site of the erythropoietin receptor (pTyr425, 69 μM).

The capacity of Tyr1077 to substitute for the mutated Tyr985 provides an explanation for the Tyr985-independent attenuation of leptin signaling observed by Dunn *et al*. [25]. Furthermore, this role for Tyr1077 may also explain why mice homozygous for a knock-in mutation of Tyr985 LEPRb display a relatively weak phenotype [50], as compared to mice with the corresponding mutation in the IL-6 cytokine family receptor signaling subunit glycoprotein 130 (gp130). The disruption of the negative feedback on gp130-dependent STAT signaling causes STAT3 hyperactivation, and these mice develop a broad spectrum of hematopoietic abnormalities, including splenomegaly, lymphadenopathy, thrombocytosis and autoimmune arthritis [51-54]. In contrast, although homozygous *Lepr*^*Leu985 *^mice display a phenotype of increased leptin sensitivity, the effect of this mutation was only significant in females and under conditions of high fat diet [50]. This mild phenotype is likely due to the negative feedback regulation through Tyr1077, which partially compensates for the mutation of Tyr985.

### SOCS1 inhibits LEPRb signaling

We have shown by direct comparisons that SOCS1 inhibits LEPRb→STAT3 signaling as potently as SOCS3. Furthermore, our results challenge the prevailing view that SOCS1 inhibits JAK kinases by directly binding to the activation loop, independent of receptor phosphotyrosine motifs [33,38,55]. Even at low expression levels, SOCS1 inhibited wild type LEPRb more efficiently than LEPRb-FYY and LEPRb-FFY (Figure 4C). This is not the first report that SOCS1 requires binding to a receptor chain tyrosine for its inhibitory effect. The inhibition of interferon-γ signaling by SOCS1 has been shown to depend strictly on the presence of Tyr441 in the interferon-γ receptor subunit 1 [56]. A similar observation has been reported for the G-CSF receptor in which Tyr729 is required for the inhibitory action of both SOCS1 and SOCS3 [57]. This result was challenged by a later study in which only the inhibitory effect of SOCS3 but not SOCS1 depended on the presence of Tyr729 [58]. However, these seemingly conflicting data may be attributed to the different cell types used and/or to varying levels of SOCS overexpression. Because high levels of protein expression can conceal potential differences in the avidity of binding sites, we took care to study the effects of the SOCS proteins on LEPRb at expression levels that cause only partial inhibition of reporter gene activity. We conclude from our data that the inhibitory effect of SOCS1 on LEPRb in HepG2 cells is strongly enhanced by the presence of the Tyr985 phosphotyrosine motif. SOCS1 is less dependent than SOCS3 on this binding site (Figure 4), consistent with the higher affinity of JAK2 for the SOCS1 *vs*. SOCS3 SH2 domain [35]. Notably, the requirement of docking sites for the actions of SOCS1 and SOCS3 may differ between different cytokine receptors. A recent study has revealed that the feedback inhibition of the oncostatin M receptor by SOCS3 was independent of phosphotyrosine motifs in the receptor chain, whereas the same levels SOCS3 did not inhibit gp130 in the absence of Tyr759 [59]. Hence, differences in requirement of recruitment sites, in addition to differences in the induction by various cytokines and different kinetics of induction [60], critically determine the modulatory effects of SOCS1 and SOCS3 on cytokine receptor signaling. Further analyses will be necessary to characterize the binding of SOCS1 to pTyr985 and reveal the physiological consequence of this interaction for the function of LEPRb.

The inhibitory effect of SOCS1 on LEPRb signaling has not previously been analyzed as a mechanism of leptin resistance, because leptin does not induce expression of SOCS1 ([39], Figure 3 of the present study). However, SOCS1 may inhibit LEPRb as a consequence of crosstalk between signaling pathways (*e.g*. IL-1β or IFNγ) rather than as a feedback mechanism. Interestingly, SOCS1 has been reported to be upregulated in adipose tissue in two different models of obesity associated with leptin resistance [61]. In the arcuate nucleus, steroid hormone dependent upregulation of SOCS1 contributes to prolactin resistance in late pregnancy [62], and may also contribute to the reduced leptin responsiveness in pregnancy. It remains to be determined whether upregulation of SOCS1 by other pathways plays a part in central or peripheral leptin resistance.

### JAK inhibition

Cytokine receptors are subject to downregulation by a variety of mechanisms, such as internalization, dephosphorylation and degradation. Here we have focused on the mechanism by which STAT activation through LEPRb is quickly attenuated within two hours of continuous leptin stimulation. As previously observed in CHO cells [39], LEPRb surface expression was not significantly reduced after two hours (Figure 2A). This result is consistent with the previous observation that presence or the absence of leptin did not alter the internalization rate of LEPRb [63]. Therefore, we asked whether the STAT phosphorylating activity of the receptor complex was altered after two hours of leptin treatment. Indeed, we observed markedly reduced activity of the JAK kinase associated with wild type LEPRb but not LEPRb-FFY. Our experimental approach allowed us to exclude several other potential mechanisms of receptor inactivation. (i) The receptor-associated JAK molecules were not dephosphorylated at this time point, excluding the possibility that tyrosine phosphatases, in particular PTP1B or SHP-2, are responsible for the fast inactivation of the JAK kinases. This result implies that the phosphorylation state of the JAKs is not a reliable measure of the catalytic activity, at least when the inactivation kinetics is concerned. (ii) The fact that the amounts of the receptor chain and the JAK kinase were not reduced after 2 h of leptin treatment suggests that the attenuation of LEPRb signaling is not a consequence of SOCS- triggered degradation of the receptor or the associated kinase [32,55]. Moreover, termination of STAT phosphorylation is not caused by the dissociation of the JAK molecules from the receptor complex, a mechanism previously described for the receptors for erythropoietin, thrombopoietin and growth hormone [64]. (iii) The direct measurement of the reduced JAK activity in the isolated receptor complex excludes the possibility that the decrease of STAT tyrosine phosphorylation is exclusively determined by the rate of STAT dephosphorylation (*e.g*. by TC-PTP [65]). The dephosphorylation of STAT1 and STAT3 must occur rapidly to allow for the fast termination of leptin signaling, but the downregulation of JAK activity is required to achieve the net reduction of STAT tyrosine phosphorylation and activation.

## Conclusions

Taken together, we conclude from the available evidence and our present results that the first step in the attenuation of LEPRb→STAT3 signaling in cells chronically stimulated by leptin occurs through the fast upregulation of the negative feedback regulator SOCS3. SOCS3 is recruited to either pTyr985 or pTyr1077 in the receptor chain and inhibits the receptor-associated JAK molecules by direct interaction of its kinase inhibitory region (KIR) with the activation loop of the kinase. The next step is putatively the dephosphorylation of the JAK molecules and/or the receptor, which is a fast event in some cell systems (*e.g*. Hek293 [25]) but was separable from the initial inhibition of JAK activity in our experiments. Other mechanisms of negative regulation may play a role at later stages and contribute to the state of reduced leptin responsiveness induced by chronic leptin stimulation.

## Methods

### Cytokines and antibodies

Recombinant murine leptin was purchased from PeproTech (London, UK) and recombinant human IL-1β from Roche Molecular Biochemicals (Mannheim, Germany). The following primary antibodies were used: polyclonal rabbit antibodies directed against p(Tyr701)-STAT1, p(Tyr705)-STAT3, STAT1, STAT3 (Cell Signaling Technology, Beverly, MA), p(Tyr1007/Tyr1008)-JAK2 (BioSource Technologies, Camarillo, CA) and SOCS3 (H-103, Santa Cruz Biotechnology, Santa Cruz, CA); goat anti mouse LEPR from R&D Systems (Minneapolis, MN), mouse monoclonal FLAG-M2 from Sigma-Aldrich (Taufkirchen, Germany), secondary horseradish peroxidase-labeled antibodies from Pierce Chemical Co. (Rockford, IL), and Dianova (Hamburg, Germany). Detection of immunoprecipitated SOCS3 was performed with biotinylated SOCS3-specific antibodies (M20, Santa Cruz Biotechnology) and HRP-coupled anti-biotin antibody (New England Biolabs, Ipswich, MA).

### Cell culture and transient transfections

Rat RINm5F insulinoma cells and HepG2 hepatoma cells were grown in RPMI 1640 medium with L-glutamine, 10% (v/v) fetal bovine serum, 100 units/ml penicillin, and 100 mg/ml streptomycin at 37°C and 5% CO_2_. HIT-T15 hamster insulinoma cells were cultivated under the same conditions except that 5% horse serum was added to the medium. Transient transfections were performed by the polyethyleneimine method (Jet-PEI reagent; Polyplus-Transfection, Illkirch, France). The RINm5F cell lines stably expressing WT or mutant LEPRb have been described previously [43,44]. The different LEPRb constructs have been confirmed to differ by less than 2.2-fold in their surface expression [44].

### Binding assay

RINm5F cells expressing WT LEPRb in 6-well plates (500,000 cells per well) were serum-starved for 4 h before they were stimulated with leptin (20 ng/ml). Control cells were prepared identically but were not treated with leptin. After 2 h, cells were washed with ice-cold phosphate buffered saline (PBS) and then with ice-cold acetic acid (0.5 M NaCl, 0.2 M acetic acid, pH 2.3) to remove receptor-bound leptin [66]. After three additional washes with PBS, cell surface expression of LEPRb was measured using a mouse leptin-secreted alkaline phosphatase (SEAP) chimeric protein [47]. Bound phosphatase activity was determined by chemoluminescence using the CSPD substrate. Nonspecific binding was determined by incubating parallel cultures with leptin-SEAP in the presence of an excess of unlabeled leptin (2 μg/ml). This value was subtracted from previous total and accounted for ~20% of total binding.

### Reporter gene assays

HIT-T15 cells on six-well plates (350.000 cells per well) were transfected with 0.3 μg of pMET7-LEPRb expression plasmids [47] along with 0.75 μg each of a STAT3-responsive luciferase reporter construct containing the promoter region -215 to +6 of the rat α_2 _macroglobulin gene (pGL3α_2_M-215Luc; [67]) and β-galactosidase reporter control plasmid pSVβ-gal (Promega). HepG2 cells were plated at a density of 150.000/well and were transfected with 0.15 μg of pMET7-LEPRb. When indicated, variable amounts of expression plasmids pEF-SOCS1 or pEF-SOCS3 (kindly provided by D. Hilton, WEHI, Parkville, Australia) or knockdown plasmids for human SOCS1 (pSM2 vector, target sequence TGTGGAAAGGACGAAACACC; Openbiosystems clone v2HS-23987) or SOCS3 (pSUPER-SOCS3, gift of Fred Schaper, Institute of Biochemistry, Aachen, Germany, [68]) were cotransfected. Control wells for shRNA experiments were cotransfected with empty vector (pSUPER) or nonsilencing pSM2 construct. Thirty hours after transfection (54 h in shRNA experiments), cells were stimulated with 100 ng/ml leptin for 16 h in serum-free medium. Luciferase activities were determined from duplicate wells and were normalized to β-galactosidase activities.

### Northern blot analysis

RINm5F-LEPRb cells grown to 80% confluence were incubated in serum-free medium for 20 h before they were stimulated with leptin (100 ng/ml) or left unstimulated for variable times. Total RNA was isolated using the RNeasy Midi Kit (Qiagen, Hilden, Germany), and samples (10 μg) of total RNA were separated by denaturing formaldehyde electrophoresis on 1% (w/v) agarose gels and transferred by capillary blot onto nylon membranes (Hybond N+; Amersham, Freiburg, Germany). ^32^P-labeled probes were generated from the inserts of cDNA clones pEF-SOCS1 and pEF-SOCS3 by random oligonucleotide priming. Blots were rehybridized with additional cDNA probes following probe removal with 0.5% SDS at 90-100°C.

### Immunoprecipitation and Western blot analysis

For immunoprecipitation of LEPRb receptor complexes, RINm5F-LEPRb cells from a near-confluent 85-mm plate were lysed on ice for 30 min in 1 ml of native lysis buffer (20 mM Tris-HCl pH 7,5; 150 mM NaCl, 1% (v/v) Brij97, 1 mM EDTA, 10 mM NaF, 1 mM Na_3_VO_4_, 1 mM PMSF and 5 μg/ml each of aprotinin and leupeptin). Insoluble material was removed by centrifugation (3 min at 14,000 rpm) before 12 μl of agarose-coupled c-myc antibody (monoclonal 9E11, Santa Cruz Biotechnology) was added to the supernatant and incubated for 4 h at 4°C. For detection of SOCS3, immunoprecipitations were performed with 1% Nonidet P-40 instead of Brij97 in the lysis buffer and 1 μg of SOCS3 antibody per 1 ml of lysate. The resin was washed twice with lysis buffer and twice with lysis buffer without detergent, and the bound proteins were eluted with denaturing gel loading buffer.

Total cellular lysates for the detection of STAT3 tyrosyl phosphorylation were prepared using denaturing lysis buffer (1% SDS, 20 mM Tris-Cl pH 7.4) and incubation in a boiling water bath for 5 min. For Western blot analysis, samples were separated by SDS-PAGE (8% gels), blotted onto nitrocellulose, and positive signals were revealed by chemiluminescence using the indicated primary antibodies and horseradish peroxidase- labeled secondary antibody. Blots were re-probed after removing the primary antibody by incubating the membrane in stripping buffer (2% SDS, 50 mM Tris-HCl, 150 mM NaCl, pH 7.4, 100 mM 2-mercaptoethanol) for 20-30 min at room temperature.

### Assay of JAK kinase in LEPRb complexes

LEPRb complexes were immunoprecipitated using agarose coupled c-myc antibody (described above) and washed twice with kinase buffer (10 mM HEPES pH 7.6, 50 mM NaCl, 5 mM MgCl_2_, 5 mM MnCl_2_, 0.4 mM DTT, 1 mM Na_3_VO_4_, 5 μg/ml aprotinin, 5 μg/ml leupeptin). For the assay, 2.2 μg of recombinant STAT1 and 28 nmol ATP in 30 μl kinase buffer were added to the pellets (estimated final concentrations 63 ng/μl STAT1 and 0.8 mM ATP). After incubation at 30°C for 45 min, supernatants were removed for Western blot analysis of STAT1 tyrosine phosphorylation. The resin with the bound receptor complexes was washed twice with wash buffer (20 mM Tris-HCl pH 7.5, 150 mM NaCl), eluted, and subjected to Western blot analysis.

### Statistical testing

Significance of differences in pairwise comparisons was analyzed using Student's t-test. Welch's t-test was used in case of unequal variances.

## Authors' contributions

HK designed and carried out all of the experiments not attributed to other authors and analysed the results. JZ generated the RINm5F cells expressing LEPRb constructs and analyzed the kinetics of STAT3 phosphorylation. PH carried out the Northern blotting and participated in and coordinated the construction of vectors and cell lines. SBB performed a large part of the luciferase assays. WB conceived of the study, and participated in its design and coordination and wrote the manuscript. All authors read and approved the final manuscript.

## Supplementary Material

Additional file 1 **Supplementary figures**. This PDF file contains 2 supplementary figures which show the time course of leptin-induced STAT3 phosphorylation in HIT-T15 insulinoma cells (Figure S1) and the inhibitory effect in reporter gene assays and the expression level of SOCS3 in HIT-T15 cell (Figure S2). Click here for file
