# Supplementary material for: Mechanism of attenuation of leptin signaling under chronic ligand stimulation
Source: BMC Biochem. 2010 Jan 8;11:2. doi: 10.1186/1471-2091-11-2 (PMC2821298; doi:10.1186/1471-2091-11-2)
Supplement: Additional file 1 — Supplementary figures. This PDF file contains 2 supplementary figures which show the time course of leptin-induced STAT3 phosphorylation in HIT-T15 insulinoma cells (Figure S1) and the inhibitory effect in reporter gene assays and the expression level of SOCS3 in HIT-T15 cell (Figure S2). [file 1471-2091-11-2-S1.PDF]

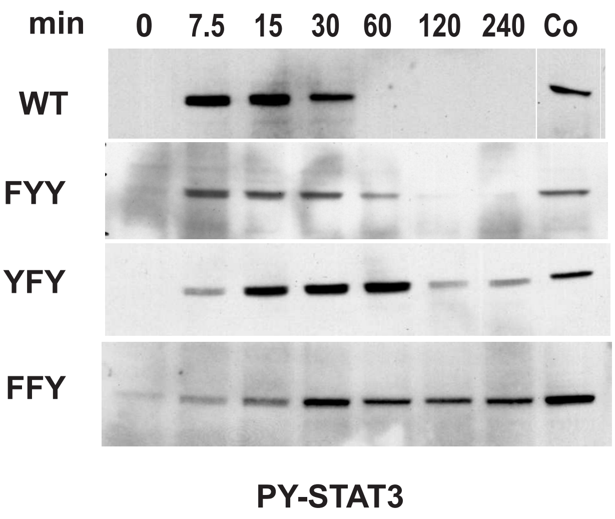

Figure S1: Time course of leptin-induced activation STAT3 in HIT-T15 insulinoma cells. - HIT-T15 cells were transiently transfected with expression plasmids for wild type LEPRb (WT) or the indicated point mutants (FYY, YFY, FFY). The following day, cells were stimulated with leptin (100 ng/ml), and the time course of leptin-induced STAT3 phosphorylation was followed by Western blot analysis with an activation-state specific antibody directed against pTyr705. The cell culture medium from 240-min leptin treated cells was transferred to naive cells for 15 min (Co) in order to exclude the possibility that leptin was degraded or inactivated in course of the incubation.

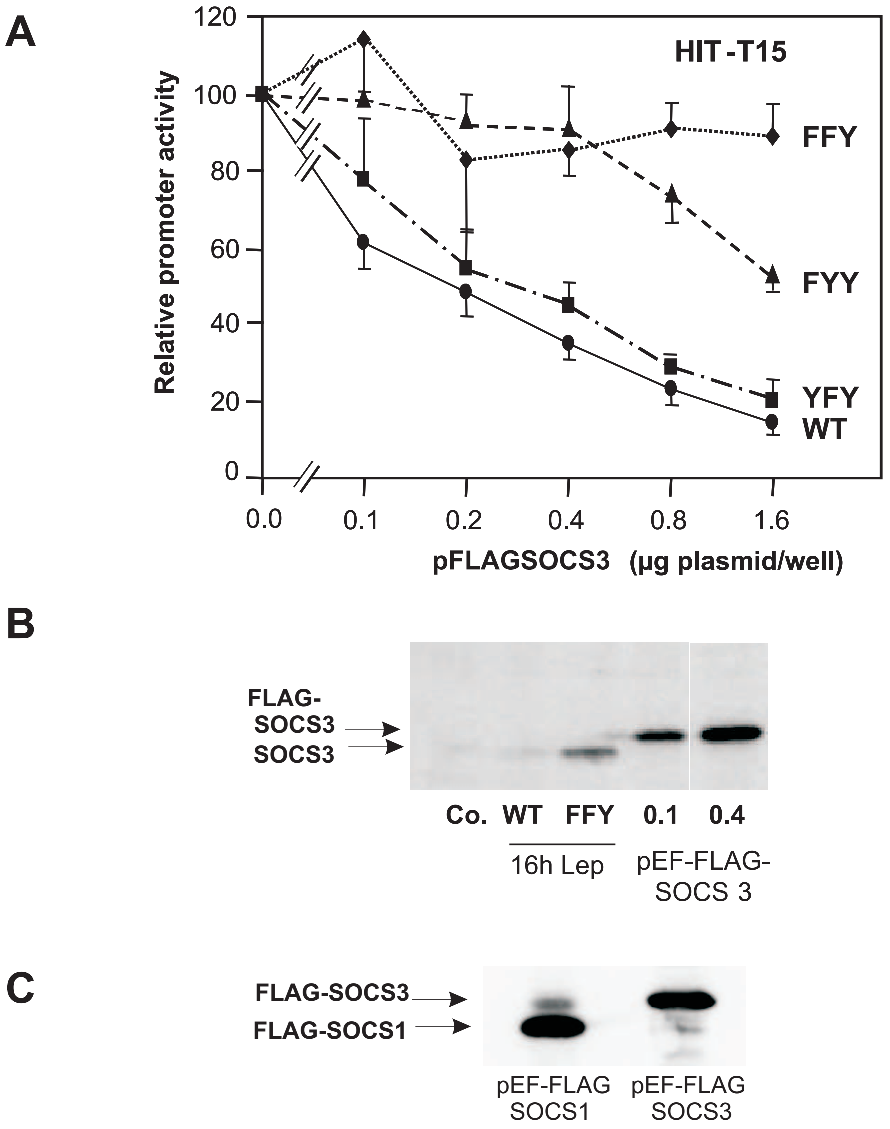

Figure S2

A: Effect of SOCS3 on reporter gene induction in HIT-T15 cells

HIT-T15 cells were transfected with pGL3x2M-215Luc and vectors for the indicated LEPRb point mutants along with varying amounts of expression plasmids for SOCS3. Twenty-four hours after transfection, cells were treated with leptin (100 ng/ml) for 20 h. Luciferase activities were normalized to β-galactosidase activities and are shown relative to those in cells expressing no exogenous SOCS3 (100%). Data points represent means and S.D. values from n=3 independent experiments.

B and C: Expression levels of SOCS1 and SOCS3.

HIT-T15 cells were transfected with expression plasmids for wild type (WT) or mutant LEPRb (FFY) as indicated or with pEF-FLAG-SOCS3 (0.1 µg or 0.4 µg/well). **B,** Levels of endogenous SOCS3 in leptin-treated cells (20 h,100 ng/ml) or overexpressed FLAG-SOCS3 were determined by immunoprecipitation and successive Western blot analysis with SOCS3-specific antibody (B).

C, Lysates of HIT-T15 cells transfected with pEF-FLAG-SOCS1 or -SOCS3 (400 ng/well) were analyzed by Western blotting with FLAG-specific antibody.
